# Neurocognitive concerns and neurocognitive impairment in long-term survivors of oropharyngeal cancer post (chemo)radiotherapy

**DOI:** 10.1007/s11764-025-01872-8

**Published:** 2025-08-02

**Authors:** Zsuzsanna Iyizoba-Ebozue, Robin Prestwich, Maryse Luijendijk, Emma Nicklin, Chinyereugo Umemneku-Chikere, Stuart Currie, James Price, Sarah R. Brown, Emma Hall, John Lilley, Matthew Lowe, Marcus Tyyger, David J. Thomson, Finbar Slevin, Louise Murray, Florien Boele

**Affiliations:** 1 Department of Clinical Oncology, Leeds Cancer Centre, St. James’s University Hospital, Beckett St, Level 4, Bexley Wing, Leeds, LS9 7TF UK; 2 Leeds Institute of Medical Research, University of Leeds, Leeds, UK; 3 Division of Psychosocial Research and Epidemiology, Netherlands Cancer Institute, Amsterdam, The Netherlands; 4 Center for Review and Dissemination, University of York, York, UK; 5 Department of Neuroradiology, Leeds Teaching Hospitals NHS Trust, Leeds, UK; 6 Department of Clinical Oncology, The Christie NHS Foundation Trust, Manchester, UK; 7 Leeds Cancer Research UK Clinical Trials Unit, Leeds Institute of Clinical Trials Research, Leeds, UK; 8 Clinical Trials and Statistics Unit, Institute of Cancer Research, London, UK; 9 Medical Physics and Engineering, Leeds Teaching Hospitals NHS Trust, Leeds, UK; 10 Christie Medical Physics and Engineering, The Christie NHS Foundation Trust, Manchester, UK; 11 Faculty of Biology, Medicine and Health, Manchester Academic Health Sciences Centre, The University of Manchester, Manchester, UK; 12 Faculty of Medicine and Health, Leeds Institute of Health Sciences, University of Leeds, Leeds, UK

**Keywords:** Neurocognition, Radiotherapy, Oropharyngeal cancers, Amsterdam Cognition Scan, Survivorship

## Abstract

**Background:**

Little is known about neurocognitive concerns (NCC) and neurocognitive function (NCF) in survivors of oropharyngeal cancer (OPC) following (chemo)radiotherapy.

**Methods:**

A multicentre cross-sectional study was conducted in non-surgically treated OPC. Using the Medical Outcomes Study Cognitive Functioning Scale (MOS-Cog), NCC were classed as having concerns at least some of the time (MOS-Cog score of ≤ 60 across all items). Patients were also invited to complete an online cognitive test battery (Amsterdam Cognition Scan; ACS) to assess NCF. Using norm scores from 708 individuals from the UK general population, significant NCF impairment was defined as *Z* score ≤  − 2 in one domain or ≤  − 1.5 in at least two domains. Regression analysis was performed to identify factors associated with NCC/NCF.

**Results:**

Three hundred thirty-eight patients treated for OPC, with median age at treatment of 58.6 years and median follow-up of 6 years (IQR 4–8) post-treatment, were recruited. NCC were present in 14.8% of patients. Amongst 93 patients who completed the ACS, 33% demonstrated significant impairment in NCF. Memory and attention were the most affected domains in NCC and NCF. On multivariable regression analysis, mental fatigue, mood, general fatigue, and physical fatigue significantly predicted NCC. Multivariate analysis revealed a small but statistically significant negative association between mood and NCF. No statistically significant correlations were found between NCC/NCF impairment and radiation dose received by the posterior fossa.

**Conclusion:**

NCC and NCF impairment affects a substantial proportion of long-term survivors of OPC. Assessment of NCC and NCF is essential for a comprehensive understanding of cognitive health in this population.

**Implications for cancer survivors:**

This study highlights that a substantial proportion of long-term oropharyngeal cancer survivors experience both subjective NCC and objective NCF impairment. The findings underscore the need for neurocognitive screening and a multidisciplinary approach to survivorship care, incorporating psychological and cognitive support alongside traditional follow-up.

**Supplementary Information:**

The online version contains supplementary material available at https://doi.org/10.1007/s11764-025-01872-8.

## Introduction

Late toxicity is a major concern for survivors of head and neck cancer (HNC) [1]. Neurocognitive issues have been identified as a key rehabilitation need for HNC survivors [2]. Yet neurocognitive toxicity is not currently included in UK HNC consent guidance due to uncertainties around the frequency and severity of issues [3]. A systematic review from our group exploring the neurocognitive outcomes of HNC following (chemo)radiotherapy ((C)RT) suggested inferior neurocognitive outcomes compared with controls, although the vast majority of studies focussed on nasopharyngeal cancers (NPC) for which substantial radiotherapy doses are delivered to the skull base/adjacent central nervous system (CNS) structures [4]. However, studies to date are lacking information on neurocognitive outcomes specific to oropharyngeal cancer (OPC) survivors, who have a favourable prognosis and are often treated with curative intent.

Neurocognitive function (NCF) is a performance outcome, assessed by means of neuropsychological tests [5]. Self-reported neurocognitive concerns (NCC), by contrast, are assessed by means of questionnaires and reflect whether a person considers that they experience cognitive issues in everyday life [6, 7]. NCC and NCF are multidimensional constructs, which are interconnected but do not fully overlap [8–10]. Both reflect the impact of late toxicity on patients’ well-being, either from a subjective point of view (NCC) or by test performance (NCF). The impact of radiation dose on cranial structures has been extensively studied in the context of brain tumours [11–13] yet, there has been considerably less research in HNC, where some radiotherapy dose is also delivered to the brain [14].

Therefore, this study aimed to evaluate the prevalence of NCC and NCF impairment in patients treated for OPC from 2 years post-radiotherapy. Furthermore, we aimed to explore the relationship between NCC/NCF and patient and treatment factors, including associations with radiotherapy dose to the posterior fossa. The findings of this study could inform follow-up care and identify survivor support needs, potentially improving patient quality of life (QoL).

## Methodology

### Study design

This study is part of a multicentre mixed-methods research project (ROC-oN: Radiotherapy for Oropharyngeal Cancer and impact on Neurocognition), approved by the West Midlands Research Ethics Committee in October 2022 (22/WM/0207). This report focuses on neurocognitive outcomes, adopting a cross-sectional design using quantitative data and comparing patients to matched normative control data. Previous publications have addressed ROC-oN fatigue outcomes [15] and interview findings [16].

### Patients and procedures

OPC survivors treated between 2010 and 2020 were recruited from two major hospitals in England: the Leeds Teaching Hospitals NHS Trust (LTHT) and The Christie NHS Foundation Trust (The Christie). Patient eligibility criteria were as follows: ≥ 18 years old at time of treatment, having undergone radiotherapy ± chemotherapy with curative intent; ≥ 24 months follow-up and disease free at time of recruitment. Patients were ineligible if they received upfront or salvage surgery.

In LTHT, the standard dose for definitive radiotherapy was 70Gray (Gy) in 35 fractions or 65 Gy in 30 fractions. The Christie used 66 Gy in 30 fractions. Treatment transitioned from 3-dimensional planning (3DP) to intensity-modulated therapy (IMRT) in 2011 and then volumetric-modulated arc therapy (VMAT) as treatment techniques evolved. Concurrent chemotherapy was cisplatin (100 mg/m^2^, three-weekly) or carboplatin (AUC5) for those with renal impairment [17].

Potential patients were identified and screened from institutional databases. They received a postal invitation to complete questionnaires and an online cognitive test battery, at a single time point. Written consent was obtained.

### Outcome measures

#### Sociodemographic and clinical information

A structured questionnaire was used to collect sociodemographic data. Medical information was extracted from electronic clinical records.

#### Patient-reported outcomes

*NCC* were assessed with the Medical Outcomes Study Cognitive Functioning Scale (MOS-Cog), a six-item questionnaire with good internal consistency [18]. Responses on the six-point MOS-Cog scale ranged from ‘all of the time’ (1) to ‘none of the time’ (6) [19],  were converted to a 0–100 scale and averaged for a total score (range 0–100) [20]. Lower scores indicated greater concerns.

*Fatigue* was assessed with the Multidimensional Fatigue Inventory (MFI) [21], a validated 20-item tool. Scored on a 5-point Likert scale, it produces five domain scores (score range 4–20): general fatigue, physical fatigue, reduced activity, mental fatigue and reduced motivation. Higher scores indicate a greater burden of fatigue.

*Mood* was measured with the Profile of Mood States Short Form (POMS-SF) [22], a validated 37-item questionnaire with items rated on a 5-point Likert scale. It has six subscales: anxiety, depression, anger, fatigue, confusion, and vigour. The total Mood Disturbance (TMD) score is calculated by adding all anxiety, depression, fatigue and confusion scores and subtracting vigour scores.

#### Cognitive testing

*NCF* was evaluated with the UK English version of a comprehensive, online unsupervised neuropsychological test battery: the Amsterdam Cognition Scan (ACS). Table 1 shows specific tests and domains assessed [23].

**Table 1:** Summary of the Amsterdam Cognition Scan, relating the test to the outcome measure and to the neurocognitive domain assessed

ACS test	Outcome measure	Neurocognitive domain assessed
Connect the dots I and II	Completion time in seconds (I and II)	Processing speed Attention and working memory
Wordlist learning	Total number of correct words (trials 1 to 5)	Learning and memory
Reaction speed	Mean reaction time in seconds	Processing speed
Place the beads	Total number of extra moves	Executive functioning
Box tapping	Total number of correctly repeated sequences	Attention and working memory
Fill the grid	Completion time in seconds	Motor functioning
Word list recall and recognition	Total number of correct words, free recall and recognition	Learning and memory
Digit sequence I and II	Total number of correctly repeated sequence forward and backward	Attention Attention and working memory

In a separate study, approved by the University of Leeds Faculty of Medicine and Health Research Ethics Committee (MREC 20–047), we gathered UK normative data for the ACS from a cancer-free norm group. These normative data were obtained from a sample of 708 adults who completed the British ACS and had no history of a cancer diagnosis. The norms, demographically adjusted for age, sex, educational level, and computer use, were established using regression-based norming following the exact same procedures as for the Dutch ACS norms [24]. Supplementary 1 highlights the normative study procedure, eligibility criteria, characteristics of the normative sample, and methods for establishing the norms.

#### Exploratory dosimetric work

For 144 patients treated in LTHT, radiotherapy plans were reviewed to explore potential relationships between radiation dose to brainstem and anterior/posterior cerebellum and NCC and NCF. These central nervous system regions of interest (CNS-ROIs) were manually delineated by oncologist (ZIE) and contours quality assured by a neuroradiologist (SC). These brain substructures were chosen because they were found to receive the highest radiotherapy doses during OPC treatment [15]. Other brain substructures received only very low radiotherapy doses (< 1 Gy) so were not considered relevant for further evaluation in this context.

### Data analysis

Patient sociodemographic and clinical data were analysed descriptively using STATA 18.0. Scores for patient-reported outcomes (MOS-Cog, MFI, POMS-SF) were generated following scoring manuals. There is no widely accepted minimal clinically important difference (MCID) for the MOS-Cog. Therefore, to aid clinical utility and to detect significant complaints, patients were classified as having ‘no cognitive concerns’ (MOS-Cog scores > 60 in all items, indicating little to no concerns) [19] or having ‘cognitive concerns’ (MOS-Cog scores ≤ 60 in all items, indicating concerns at least some of the time). This threshold for ‘cognitive concerns’ is more stringent than the definition used by Karr et al. [19], where NCC in patients with dementia was defined as MOS-Cog ≤ 60 in two items. This mitigates overestimating NCC. Patients’ raw ACS test scores were converted to standardised *Z*-scores, adjusted for age, sex, educational level, and computer use based on the norms obtained from the British cancer-free reference group. As per recommendations of the International Cognition and Cancer Task Force (ICCTF), clinically significant NCF impairment was considered present if a patient had a *Z*-score of ≤  − 2 on a single domain or a *Z*-score of ≤  − 1.5 (− 1.5 to − 1.9) in two or more domains [25, 26]. We hypothesised that radiotherapy or chemoradiotherapy for OPC would have a negative impact on both objective and self-reported cognitive function.

The relationship between NCC/NCF, sociodemographic, treatment factors, and other patient outcomes was examined. Analyses were structured to maximise information derived from the data and align with both exploratory and clinical objectives of this study. Firstly, multiple linear regression was conducted to examine the relationship between potential predictors and the total MOS-Cog score, enabling identification of trends and effect sizes. Secondly, a logistic regression was then conducted post hoc on dichotomised data (presence or absence of cognitive concerns; NCC) to evaluate predictors in the context of binary outcomes relevant to clinical decision-making and intervention planning. Similarly, multivariate linear regression was performed using ACS *Z*-scores as continuous variables, focusing on domains where ≥ 10% of patients demonstrated a deficit (*Z*-score <  − 2). Subsequently, post hoc logistic regression was conducted to evaluate the likelihood of clinically significant NCF impairment, defined as its presence or absence.

Key variables included in the selection modelling were based on evidence from modelling studies focused on cognitive outcomes in patients with brain tumours [27] and clinical relevance. Variables (age at treatment, sex, education, smoking, radiation dose, chemotherapy, physical fatigue, reduced activity, mental fatigue, general fatigue, reduced motivation, total mood and follow-up time) were analysed using stepwise regression, employing both forward and backward selection methods to refine the model (supplementary 3). The model with the least Akaike information Criterion (AIC) from the stepwise selection was selected and carried forward. The final model included variables with statistical significance (*p* < 0.05) alongside clinical opinions on the relevance of the variables (supplementary 3). Multicollinearity between the variables was evaluated, and variables were excluded if the variance inflation factor (VIF) was ≥ 5. Coefficients and odds ratio (OR) with 95% confidence intervals, as well as *p*-values, were reported.

In addition, for ACS domains where ≥ 10% of participants demonstrated a deficit, Spearman’s correlations with ACS *Z*-scores for that test and the corresponding MOS-Cog item (item score) evaluating the same domain were performed.

Finally, exploratory analysis of potential relationships between radiation dose to brainstem and anterior/posterior cerebellum and NCC and NCF was conducted. In 144 participants treated in LTHT, radiotherapy plans were imported into RayStation (RaySearch Laboratories, Stockholm, Sweden version 11DaK) and MRI co-registration was performed where possible, using 2 mm T1 contrast-enhanced slices. The CNS regions of interest (CNS-ROI) were contoured by an oncologist (ZIE) and quality assured by a neuroradiologist (SC). Dose distributions were converted to equivalent dose in 2 Gy per fraction, using an alpha–beta value of 3 Gy [28]. Near-maximum dose (D1cc), mean dose (Dmean), and the volume receiving 10–30 Gy (V10-V30 in 5 Gy increments with 95% CI) for each substructure were extracted. To address potential multicollinearity, a composite structure called the ‘posterior fossa’ (comprising the brainstem, anterior and posterior cerebellum) was introduced for analysis. Correlations between CNS-ROI doses and (i) NCF (focusing on domains where ≥ 10% of patients demonstrated a deficit [Z-score <  − 2]) and (ii) NCC were investigated.

Missing data were below 10% for self-reported outcomes (3% for MOS-Cog, 4% for MFI and 9% of POM-SF). The ACS was not completed by 73% of the sample; radiotherapy dosimetry was not evaluated for patients recruited from Manchester (57%). Missing data were not confirmed missing completely at random (MCAR), and therefore imputation was not used to prevent introducing potential bias or inaccuracies that could arise from systematic patterns in the missing data.

## Results

A total of 855 patients treated for OPC were invited, of whom 349 enrolled in the study. Of these individuals, 338 completed the MOS-Cog and were taken forward into analysis. The ACS was completed by 93 patients. Patient demographics, disease and treatment details are provided in Table 2. Median interval from the completion of radiotherapy was 6 years (IQR 4–8).

**Table 2:** Demographics of participants who completed the Medical Outcomes Study Cognitive Functioning Scale and Amsterdam Cognition Scan

	Study sample (completing MOS-Cog *n* = 338)	Study sample (completing ACS *n* = 93)
Age		
Age at diagnosis: mean (SD)	58.6 (8.5) yrs	56.4 (7.7) yrs
Age at study recruitment: mean (SD)	64.5 (8.6) yrs	61.6 (7.7) yrs
Sex		
Male	248 (73.4%)	71 (76.3%)
Female	90 (26.6%)	22 (23.7%)
Level of education		
No formal qualification/below secondary education	58 (17.2%)	5 (5.4%)
Completed secondary education but below university level	192 (56.8%)	54 (58.0%)
University degree or higher	79 (23.4%)	29 (31.2%)
Unknown	9 (2.6%)	5 (5.4.0%)
Current employment status		
Employed	143 (42.3%)	45 (48.3%)
Unable to work due to illness/disability	19 (5.6%)	2 (2.2%)
Unemployed	7 (2.0%)	2 (2.2%)
Retired	166 (49.1%)	41 (44.1%)
Unknown	3 (0.9%)	3 (3.2%)
Pretreatment ECOG performance status		
0	290 (85.8%)	91 (97.8%)
1	44 (13.1%)	2 (2.2%)
2	4 (1.1%)	0 (1.1%)
Smoking at time of treatment		
Current smoker	64 (18.9%)	10 (18.6%)
Ex-smoker	108 (31.9%)	31 (32.6%)
Never smoked	162 (47.9%)	52 (47.2%)
Unknown	4 (1.2%)	0 (0%)
Tumour subsite		
Tonsil	213 (63.0%)	67 (72.0%)
Base of tongue	112 (33.1%)	26 (28.0%)
Vallecula	2 (0.6%)	0 (0%)
Posterior pharyngeal wall	3 (0.9%)	0 (0%)
Soft palate	8 (2.4%)	0 (0%)
T-stage		
T1	65 (19.2%)	24 (25.8%)
T2	163 (48.2%)	38 (40.9%)
T3	61 (18.1%)	19 (20.4%)
T4	49 (14.5%)	12 (12.9%)
N-stage TNM7		
N0	53 (15.7%)	10 (10.8%)
N1	42 (12.4%)	15 (16.1%)
N2	201 (59.5%)	47 (50.5%)
N3	42 (12.4%)	21 (22.6%)
P16 status		
Positive	276 (81.7%)	81 (87.0%)
Negative	26 (7.7%)	3 (3.3%)
Unknown	36 (10.6%)	9 (9.7%)
Fractionation		
70 Gy/35#	138 (40.8%)	45 (48.4%)
65–66/30#	200 (59.2%)	48 (51.6%)
Concurrent chemotherapy	272 (80.5%)	79 (84.9%)
Radiotherapy alone	70 (19.5%)	14 (15.1%)

Tumour stage (T-stage), nodal stage (N-stage), surrogate immunohistochemical marker of oncogenic human papilloma virus (HPV) infection (P16), Eastern Cooperative Oncology Group (ECOG) performance status

### Prevalence of NCC and NCF

The MOS-Cog results (*n* = 338) are summarised in Table 3. Patients had concerns most frequently in the domains of memory and attention: 43.4% and 34.3% of patients scored ≤ 60 on MOS-Cog in these domains, respectively. In total, 50/338 (14.8%) were categorised as having ‘cognitive concerns’ (≤ 60 in all domains), and 158/338 (46.7%) were categorised as ‘no cognitive concerns’ (> 60 in all domains). Furthermore, 38.4% (130/338) had mixed scores of >/< 60 across the domains indicating a lower level of cognitive concerns.

**Table 3:** Patient reported outcomes and ACS outcomes

Patient outcomes	*N* (%)	Mean (SD)
MOS-Cog		
Total MOS-Cog		75.5 (22.5)
Reasoning (score)		
0	9 (2.6)	
20	20 (5.8)	
40	35 (10.2)	
60	48 (14.0))	
80	112 (33.4)	
100	114 (37.2)	
Forgetting (score)		
0	14 (4.1)	
20	24 (7.6)	
40	41 (12.0)	
60	37 (19.6)	
80	104 (31.0)	
100	86 (25.5)	
Attention (score)		
0	8 (2.3)	
20	26 (7.6)	
40	26 (7.6)	
60	57 (16.7)	
80	107 (31.6)	
100	114 (34.0)	
Concentration (score)		
0	7 (2.0)	
20	21 (6.2)	
40	33 (9.7)	
60	47 (13.9)	
80	118 (34.9)	
100	112 (33.1)	
Confusion (score)		
0	3 (0.8)	
20	19 (5.6)	
40	23 (6.7)	
60	32 (9.4)	
80	70 (20.6)	
100	191 (56.6)	
Slow reaction speed (score)		
0	4 (1.1)	
20	12 (3.5)	
40	32 (9.4)	
60	44 (13.0)	
80	95 (28.1)	
100	151 (44.6)	
ACS (No. patients with *Z*score ≤ − 2)		
Connect the dots I (processing speed)	8 (8.6)	
Connect the dots II (attention and working memory)	8 (8.6)	
Word list learning (learning and memory)	2 (2.2)	
Delayed recall (learning and memory)	3 (3.2)	
Reaction speed (processing speed)	2 (2.2)	
Place the bead (executive functioning)	4 (4.3)	
Box tapping (attention and working memory)	10 (10.8)	
Fill the grid (motor functioning)	0 (0)	
Digit span I (attention)	0 (0)	
Digit span II (attention and working memory)	0 (0)	
MFI		
General fatigue		11.12 (4.89)
Physical fatigue		10.43 (5.10)
Reduced activity		9.18 (4.65)
Mental fatigue		9.46 (4.72)
Reduced motivation		8.99 (4.29)
POM-SF		
Fatigue-Inertia		6.17 (5.81)
Depression-dejection		4.17 (6.38)
Anger-hostility		3.20 (5.03)
Tension-anxiety		4.55 (5.59)
Confusion-bewilderment		3.45 (4.36)
Vigour-activity		9.74 (6.24)
Total mood disturbance		11.81 (25.67)

*MOS-Cog* Medical Outcomes Study Cognitive Functioning Scale, *ACS* Amsterdam Cognition Scan, *MFI* Multidimensional Fatigue Inventory, *POMS-SF* Profile of Mood States Short Form

The ACS was completed by 93 patients. Impairments (*Z*-score of ≤  − 2 on a single domain) were mostly identified in the domains of attention and working memory (10.8% affected on box tapping and 8.6% on connect the dot II), followed by processing speed and executive function (Table 2). No clinically relevant levels of impairment were reported in patients treated for OPC in the domain of motor dexterity/functioning. Overall, NCF was classed as impaired in 31/93 (33.3%): 24/31 had *Z*-scores ≤  − 2 in at least one domain and 7/31 had *Z*-scores ≤  − 1.5 in at least two domains.

### Factors associated with NCC and NCF

Multivariate linear regression revealed significant associations between total MOS-Cog scores, mood, and fatigue domains. Mood demonstrated a negative relationship (*β* =  − 0.32 [95% CI − 0.41, − 0.24], *p* < 0.001), indicating that greater mood disturbance was linked to lower MOS-Cog scores. Mental fatigue (*β* =  − 2.59 [95% CI − 3.06, − 2.11], *p* < 0.001) and general fatigue (*β* =  − 0.75 [95% CI − 1.34, − 0.17], *p* = 0.05) also showed negative relationships, with higher fatigue corresponding to lower cognitive scores. In contrast, physical fatigue was positively related to MOS-Cog (*β* = 0.65 [95% CI 0.15, 1.15], *p* < 0.01). The model (df = 293, *p* < 0.001) explained 67% of the variance (*R*^2^ = 0.67) and had an AIC of 2428.5(supplementary 3 Table 4). No multicollinearity was detected (VIFs ranged from 1.02 to 2.83, supplementary 3 Table 7).

For NCF, box tapping was the only domain investigated due to the higher incidence of impairment in this domain. In multivariate linear regression analysis, mood showed a small but statistically significant negative association with box tapping *Z*-scores: mood: − 0.02 (95% CI − 0.04 to − 0.006), *p*-value < 0.05. Statistical measures for the model were adjusted *R*^2^ = 0.15, df = 79, *p* = 0.1 and AIC of 271.35 (supplementary 3 Table 6). No multicollinearity was detected; VIFs ranged from 1.12 to 2.45 for all variables (supplementary 3 Table 7).

On post-hoc analysis of NCC, the predictors mood (OR 1.04 [95% CI 1.02, 1.06], *p* ≤ 0.001) and mental fatigue (OR 1.31 [95% CI 1.14, 1.49] *p* ≤ 0.001) were statistically significant (supplementary 3 Table 4). Hence, patients with mental fatigue were more likely to report NCC, and worsening mood was associated with a slight increase in the likelihood of NCC. The relationship with physical and general fatigue on linear regression did not persist in the post hoc analysis. On post hoc logistic analysis of NCF (*Z*-score ≤  − 2 on box tapping), no significant associations were found (supplementary 3 Table 4).

No significant association was observed between neurocognitive change, NCC or NCF and chemotherapy exposure or time since completion of treatment in either univariate or multivariate analyses.

### Correlation between NCC and NCF

Evaluating attention and memory, no correlation was demonstrated between MOS-Cog scores for Item 2 (‘during the past month how much of the time did you forget, for example things that happened recently, where you put your things or appointments?’), item 3 (‘how much of the time during the past month did you have trouble keeping your attention on any activity for long?’) and item 4 (‘during the past month how much of the time did you have difficulty doing activities involving concentrations and thinking?’) and the box tapping *Z*-scores (*r* = 0.17 [*p* = 0.09], *r* = 0.18 [*p* = 0.08] and *r* = 0.11 [*p* = 0.26]). Hence, no association was demonstrated between subjective and objective assessment at the domain level for the domains where ≥ 10% of individuals demonstrated a NCF deficit.

### Correlation between dose and NCC/NCF

Of the three substructures evaluated (brainstem, anterior cerebellum, posterior cerebellum), the posterior cerebellum received the highest average Dmean and Dmax, 7.15 Gy and 29.7 Gy, respectively. For all three substructures (and the composite posterior fossa structure), there were no significant correlations between radiation dose (maximum dose, mean dose and lower dose bath (i.e. V10–V30; supplementary Table 2) and NCC (total Mos-Cog). Similarly, no significant correlations were observed between radiation dose and NCF (*Z*-scores) in the domains of attention and memory.

## Discussion

In this comprehensive analysis of a large cohort of OPC patients treated with definitive (chemo)radiotherapy at a median of 6 years post-treatment, 14.8% of patients reported NCC and 33% had NCF impairment. Predominantly affected domains were attention and working memory. No association was found between radiotherapy doses to the posterior fossa structures and NCF/NCC outcomes.

NCC offer insights into everyday challenges and social functioning [29]. The qualitative interviews conducted in a subset of patients (*n* = 21) revealed patients who reported NCC faced challenges in maintaining paid employment and participating in leisure activities, and also experienced social isolation, with a need for reassurance and support [16]. Mental fatigue emerged as a significant determinant of NCC on both linear and logistic regression. This is similar to results observed in patients with lymphoma and low-grade glioma patients [30, 31]. Increased perception of effort and aversion for cognitive tasks have been found to be common expressions of fatigue [32]. Mood was also associated with NCC, reinforcing evidence of strong correlations between subjective cognitive assessment and self-reported outcomes such as quality of life, anxiety, depression, fatigue, and sleep disturbance in cancer populations [9]. Physical fatigue displayed a positive relationship with the MOS-Cog score. This unexpected finding could be due to a potential compensatory mechanism in individuals experiencing fatigue, or it may reflect an underlying variable, such as the level of physical activity or fitness, that was not accounted for in the analysis. Further investigation is needed to better understand the dynamics of this relationship.

NCF is a measure of performance, encompassing various domains [33, 34]. NCF deficits in patients treated for OPC have not historically been recognised as a significant concern. However, amongst patients completing the ACS, 33% met the criteria for cognitive impairment, with issues primarily exhibited in the domains of attention and working memory. The evidence regarding the neuropsychological sequelae of radiotherapy in HNC is limited, with reported rates of cognitive deficits ranging from 32% to 56.8%, using various measurement tools, definitions of impairment and varying HNC populations [25, 35–37]. In a small HNC study, with predominantly OPC participants (*n* = 24: 21 OPC, 3 other), 25% of patients exhibited a deficit in NCF (measured objectively) in at least one cognitive domain, again with memory and attention being the most commonly affected [38]. In a longitudinal study by Zer et al., 38% of patients (*n* = 50: 42 OPC, 8 other HNC subsites) showed NCF decline at 24 months, with deficits primarily in intellectual capacity and verbal memory on objective assessment [39]. In the only other long-term study investigating NCF (measured objectively) in a heterogeneous HNC cohort (*n* = 44), worse performance was observed in episodic memory (11.4%) and speed of information processing (6.8%) [40]. Analysis within our investigation did not identify any patient- or cancer-related predictors of neurocognitive function (NCF). This could be due to limited statistical power, or NCF being influenced by a wide range of factors beyond traditional clinical variables, such as social factors, genetic predisposition and medications for example [33, 41, 42].

While existing literature suggests that chemotherapy can negatively impact cognitive function [43, 44], its specific effect on neurocognitive outcomes in patients treated for HNC has been infrequently studied, with findings to date being limited and inconsistent [39, 45]. In our exploratory analysis in this cross-sectional study, we found no significant association between chemotherapy exposure and either NCC or NCF. However, it is important to note that the majority of patients in our cohort received chemoradiotherapy, which may have limited the statistical power to detect an independent effect of chemotherapy. Furthermore, patients who did not receive chemotherapy were generally older and had poorer performance status, and thus may have been predisposed to cognitive impairment, irrespective of treatment. These factors highlight the need for further, adequately powered, and preferably longitudinal studies to better evaluate the impact of chemotherapy on neurocognition in this patient population.

Several studies have shown that NCC and NCF do not completely overlap but rather represent distinct constructs. Subjective neurocognitive impairment might not represent a true dysfunction, and standard objective neurophysiological testing might not detect subtle impairments at the domain level [46, 47]. It is also important to note that NCF evaluates performance at a single point under controlled conditions, whereas NCC captures a person’s experience of cognitive issues over a defined time period (MOS-Cog: the past month), likely better reflecting real-life environments [47]. Over time, patients may adapt to changes in NCF, leading to a reduced perception of NCC. Additionally, individual expectations regarding cognitive function may evolve at different stages of survivorship, further influencing the perception of NCC. Both NCF and NCC can impact various aspects of daily life, including employment, social functioning, and community integration. The absence of significant correlation between the MOS-Cog (NCC) and ACS (NCF) domains in our study supports the evaluation of both NCC and NCF to guide comprehensive cognitive care in OPC.

The role of neuroanatomical substructures in radiotherapy-induced NCF decline has primarily focused on the hippocampus and its function in memory [48]. However, the posterior cerebellum (cognitive cerebellum) [49, 50] and brainstem (part of the cerebello-cerebral network) [51, 52] are integral structures involved in cognitive processes. We did not, however, find an association between NCF or NCC and radiotherapy doses (max dose, mean dose, or low-dose bath) to these structures. Although IMRT may result in higher doses to the cerebellum than 3-dimensional planning (3DP) [53, 54], the average doses received by the posterior fossa structures (2.05 Gy, 1.5 Gy, 7.15 Gy to brainstem, anterior cerebellum and posterior cerebellum, respectively) were low and might be within (an as yet undefined) dose tolerance. Furthermore, doses delivered to cranial structures showed minimal variation due to the consistent use of the same radiotherapy technique (vast majority of patients received IMRT/VMAT). Consequently, the potential to establish a dose–response relationship is limited. Also, the role of vital neurological connections between the cerebellum and various cortical and subcortical regions [4, 55, 56] was not considered in this study. Voxel-based analysis may allow the full 3-dimensional dose distribution to be considered in relation to NCF without predefined CNS-ROIs biasing the analysis [57]. Other than the direct impact of radiotherapy dose, additional factors such as genetic susceptibility, lifestyle and metabolic abnormalities, anaemia, as well as pro-inflammatory cytokine activation resulting from intensive oncological treatment combinations, could all potentially contribute to NCF impairment [58].

The primary strengths of this study are threefold: (i) the inclusion of a large OPC-specific cohort, (ii) assessment of cognition through both subjective and objective measures, and (iii) utilisation of a novel unsupervised online cognitive battery with the potential to enable the collection of extensive neurocognitive performance datasets for future research. Although there could be variability in testing conditions, which may affect reliability when using an online cognitive battery, the ACS has demonstrated strong concurrent validity with traditional face-to-face cognitive assessment [23].

However, some limitations are acknowledged. Firstly, the survey response rate of 41% represents a limitation and must be acknowledged when interpreting the findings. While a minimum uptake of 50% is often considered desirable in similar studies [59], this benchmark does not account for the inherent challenges in recruiting individuals who are five or more years post-treatment and no longer engaged in routine oncological follow-up. This population is typically less accessible and may partly explain the response rate observed in this study. The modest number of patients who completed the ACS (27%) may also introduce potential selection bias; patients perceiving neurocognitive issues may have been more likely to participate, but equally those anticipating difficulty with assessment may have been more inclined to opt out.The cross-sectional design of the study inherently limits the availability of baseline NCF. This makes it difficult to determine if the observed neurocognitive outcomes are solely attributable to the treatment or could be contributed to by pre-existing cognitive impairments associated with cancer diagnosis itself [60]. Finally, the method used to define the NCC group may underestimate concerns, as it does not account for individuals who fall outside the ‘no concerns’ category but score ≤ 60 in at least one MOS-Cog domain.

## Conclusion

Our study findings suggest that neurocognitive problems are common amongst long-term survivors of OPC. While further investigation into the underlying mechanisms of cognitive change is needed, it remains essential that patients are monitored for neurocognitive effects and supported throughout their survivorship journey. Counselling and support services should be tailored to address these challenges, providing strategies to mitigate their effects on quality of life. Future research should employ longitudinal designs incorporating baseline assessment of neurocognitive health to enable more accurate evaluation of treatment-related changes.

## Supplementary Information

Below is the link to the electronic supplementary material.

ESM 1 (DOCX 95.5 KB)

## Data Availability

The data that support the findings of this study are available from the corresponding author[ZIE], upon reasonable request.
