# Supplementary material for: Neurocognitive concerns and neurocognitive impairment in long-term survivors of oropharyngeal cancer post (chemo)radiotherapy
Source: J Cancer Surviv. 2025 Aug 2;20(Suppl 1):904–15. doi: 10.1007/s11764-025-01872-8 (PMC13676501; doi:10.1007/s11764-025-01872-8)
Supplement: Supplementary file 1 [file 11764_2025_1872_MOESM1_ESM.docx]

**Supplementary -NCC and NCF outcomes in OPC post (chemo)radiotherapy**

1**. Collection and establishment of UK normative data for the ACS**

**Study participants and procedures**

Normative data for the British version of the Amsterdam Cognition Scan (ACS) was collected from a sample of native speaking British adults without cancer in the LOG IN UK (Collection of onLine cOGnitIve test Norms in a group of healthy participants from the United Kingdom) study. The study was conducted by the University of Leeds (PI: Dr Florien Boele) in collaboration with the Netherlands Cancer Institute and approved by the University of Leeds Research Ethics Committee (MREC 20-047). Participants were recruited between April and June 2022 from the online respondent panel TOLUNA ([www.toluna.com](http://www.toluna.com)) and received a modest monetary reward for their participation.

Potential respondents were approached by TOLUNA and provided a link to a questionnaire in Qualtrics ([www.qualtrics.com](http://www.qualtrics.com)). Upon opening the link, potential participants were first informed about the study procedures and then asked to provide online informed consent when willing to participate. Hereafter, participants completed several screener questions to assess their eligibility. Eligibility criteria included age of 18 years or older, English as first language, no (history of) diagnosis of cancer, use of and ability to use a computer or laptop with internet connection and sound, no neurological condition or psychiatric disorder that may affect cognitive function (e.g., stroke, dementia, mild cognitive impairment, epilepsy, multiple sclerosis, Parkinson’s, or Huntington’s disease), no use of medication for depression or anxiety (e.g., citalopram, fluoxetine, paroxetine, duloxetine, amitriptyline, or lithium), and no consumption of 6 or more units of alcohol per day. If eligible, additional questions about their sociodemographic characteristics and medical history followed. Upon completing the questionnaire, participants received a link to the ACS. The ACS consisted of 7 cognitive tests with 11 different outcome measures and also included two questionnaires on anxiety and depression (Hospital Anxiety and Depression Scale^2^) and fatigue (Multidimensional Fatigue Inventory^3^), and a question about self-reported number of hours per week spent using a computer. Combined, filling in the online questionnaire and completing the ACS took approximately 1 hour.

All participants who completed the ACS were approached to complete the ACS again six months later. These data will be reported elsewhere.

**Participant inclusion and characteristics**

The flowchart of inclusion is visualized in **Figure 1**. In total, 3540 responses were recorded. Partly completed responses were excluded due to missing demographic data (i.e., computer use). 710 respondents completed the questionnaire and ACS. Sociodemographic characteristics and medical history of the included normative sample are described in **Table 1**.

**Figure 1. Flowchart of participant recruitment in LOG IN UK**

**Table 1. Sociodemographic characteristics & medical history norm group**

|  | *N (%)*^a^ |
| --- | --- |
| **Sociodemographic characteristics** |  |
| **Age**, mean ±SD [range] | 50 ±17 [18-89] |
| **Sex**, *N* (%)  Female  Male | 381 (54)  327 (46) |
| **Education**  Low Middle High | 101 (14)  224 (32)  383 (54) |
| **Marital status** Single, never married Married or living together Divorced or separated (single)  Widowed (single)  In a relationship, not living together | 172 (24)  427 (60)  57 (8)  25 (4)  27 (4) |
| **Medical history** |  |
| **Smoking**  Yes No  **Units per day**, mean ±SD | 88 (12)  620 (88)  11 ±7 |
| **Alcohol use**  Yes No  **Units per week**, mean ±SD | 478 (68)  230 (32)  7 ±8 |
| **Dyslexia**  Yes No  Prefer not to say | 15 (2)  690 (97)  3 (0.4) |
| **Concussion/whiplash (ever)**  Concussion Whiplash Both  Prefer not to say | 36 (5)  52 (7)  18 (3)  6 (1) |
| **Transient Ischemic Attack (ever)**  Yes No Missing | 7 (1)  700 (99)  1 (0.3) |
| **Serious illness (ever)**  Yes No Prefer not to say Missing | 35 (5)  668 (94)  4 (0.6)  1 (0.1) |
| **Receiving treatment (current)**  Yes No Prefer not to say Missing | 212 (30)  493 (70)  2 (0.3)  1 (0.1) |
| **Indication current treatment**  Hypertension  High cholesterol Cardiovascular disease  Diabetes Mellitus Other Prefer not to say | 18 (3)  3 (0.4)  4 (0.6)  19 (3)  100 (14)  2 (0.3) |
| **Medication use (current)**  Yes No Missing | 310 (44)  397 (56)  1 (0.3) |
| **Medication type**  Sleeping pills  Blood thinners  Blood pressure medicines Antiplatelet medicines Cholesterol medicines Antidiabetic medicines Other Prefer not to say | 5 (0.7)  29 (4)  106 (15)  2 (0.3)  108 (15)  53 (7)  132 (19)  7 (1) |
| **Menstruation**^b^  Regularly Irregularly  No Prefer not to say **Age last menstruation**, mean ±SD | 131 (34)  28 (7)  220 (58)  2 (0.5)  48 ±7 |
| **Natural menopause**^c^  Yes No | 178 (81)  42 (19) |
| **Hormone Replacement Therapy use**^b^  Yes No Prefer not to say | 42 (11)  338 (89)  1 (0.3) |
| **Lifestyle factors** |  |
| **Sleeping problems**  Yes No  Missing  **Hours of sleep per night**^d^, mean ±SD | 171 (24)  536 (76)  1 (0.1)  7 ±1 |
| **Cognitive complaints**^e^, mean ±SD Memory issues Concentration issues | 1.3 ±1.8  1.3 ±1.9 |
| **Mediterranean diet**^f^  Yes No  Missing | 197 (39)  510 (72)  1 (0.3) |
| **Exercise (days)**^d,g^, mean ±SD | 11 ±9 |

^a^ Values indicate *N* (%), unless indicated otherwise. ^b^ Percentage relative to total number of females
^c^ Percentage relative to number of females not menstruating
^d^ Average over last month
^e^ In the past 24 hours, scale from 0 (=no issues) to 10 (=as bad as you can imagine)
^f^ Regularly eating fish, vegetables, pulses, (olive) oil and pasta; and sometimes drinking red wine
^g^ For at least 30 minutes, in such a way that your breathing rate increases

**Establishment of normative data**

Normative data were established following the exact same procedures as used for establishing norms from a Dutch normative sample for the Dutch version of the ACS [1]. Raw ACS test scores were checked for quality and outliers and invalid test scores were excluded from further analysis, as described previously [2, 3](with age groups ≤40, 40-60, >60). Normative data, demographically adjusted for age, sex, educational level, and computer use, were established using regression-based norming with Generalized Additive Models for Location, Scale and Shape (GAMLSS) [4]. GAMLSS is a flexible modeling framework that is able to capture nonlinearity, nonnormality and heteroscedasticity by modeling not only the location (the mean/median) of the test score distribution, but also the distribution’s shape and scale with parameters for variance, skewness and/or kurtosis, providing realistic and precise norms. Using GAMLSS, the test score distributions of 10 ACS outcomes were modeled as a function of the explanatory demographic variables. As in previous studies [2, 3] one outcome (Wordlist Recognition) was excluded from further analysis because of ceiling effects. In all models, the distribution’s location parameter was modeled as a function of the explanatory variables age, sex, educational level, and computer use. In addition, parameters for variance, skewness, and kurtosis were modeled as a function of age. All parameters were modeled using monotonic p-splines for age to be able to capture the non-linear effects of age on cognitive function.

Based on the estimated GAMLSS models, percentile scores were derived for the range of possible raw ACS test scores and each combination of levels of the explanatory variables. Percentiles scores converted to normalized Z-scores based on the cumulative distribution function (CDF). Upon request, norms can be provided either as an interactive, user-friendly Excel file or as look-up tables accompanied by a customized R script.

**2.Exploratory dosimetric work**

Table 2. Low dose bath to CNS-ROIs

| Low -dose bath | Brainstem  (mean cm^3^, SD) | Anterior Cerebellum  (mean cm^3^, SD) | Posterior Cerebellum  (mean cm^3^, SD) | Posterior Fossa  (mean cm^3^, SD) |
| --- | --- | --- | --- | --- |
| V10 | 6.01 (2.79) | 0.2 (0.66) | 41.13 (24.21) | 43.25(24.7) |
| V15 | 4.58 (2.46) | <0.1 | 23.25 (19.75) | 24.8(19.5) |
| V20 | 3.02 (2.22) | <0.1 | 12.50 (14.31) | 13.6(14.5) |
| V25 | 1.72 (1.67) | <0.01 | 6.12 (8.88) | 6.7 (8.9) |
| V30 | 0.77 (0.95) | <0.001 | 2.69 (4.92) | 2.8(4.7) |

**3. Regression analysis**

There were 301 patients with complete information on the variables of interest in the MOSCOG (NCC); while there were 85 patients with complete information on the variables of interest in the ACS (NCF). Variables (factors) included in the selection modelling were based on evidence from systematic reviews[5] and clinical opinion. The stepwise approach was used to help select variables that were relevant to the model by looking at model with the lowest Akaike information Criterion (AIC), and variables with statistical significance. Furthermore, clinically relevant variables based on their ability to influence cognitive outcomes, input from clinical experts and relevance in clinical settings were considered.

3.1. Moscog (NCC)

The total MOSOG score (dependent variable) is measured as a continuous outcome for the linear regression and the NCC is measures as binary outcome for the logistic regression. The independent variables included in the MOSCOG / NCC model are age at treatment, sex, education, smoking(yes or no), radiation dose, chemotherapy (yes or no), physical fatigue, reduced activity, mental fatigue, general fatigue, reduced motivation, total mood and follow up time. The coefficient of all variables included in the model before the stepwise selection approach was implemented is presented in table 3 below.

**Table 3.** Coefficient of all variables explored in NCC regression analysis

| **Multiple linear regression** | | |
| --- | --- | --- |
| **NCC** | ***Co-efficient (95%CI)*** | **P value** |
| Age at treatment | 0.22 (0.004, 0.435) | 0.0464 |
| Sex (female) | -2.62 (-6.13, 0.89) | 0.15 |
| Education | -0.013 (-0.87, 0.85) | 0.98 |
| Smoking | 0.67 (-2.47, 3.80) | 0.68 |
| Radiation dose | -0.0003 (-0.0008, 0.0002) | 0.24 |
| Chemotherapy | 4.87(-0.09, 8.97) | 0.09 |
| Physical fatigue (MFI scale) | 0.71 (0.12, 1.31) | 0.02 |
| Reduced activity (MFI scale) | -0.23 (-0.82, 0.36) | 0.44 |
| Mental fatigue(MFI scale) | -2.61 (-3.10, -2.12) | <0.00001 |
| General fatigue(MFI scale) | -0.70 (-1.29, -0.12) | 0.02 |
| Reduced motivation (MFI scale) | 0.23 (-0.42, 0.87) | 0.49 |
| Total mood disturbance (POMS-SF) | -0.33 (-0.42, -0.25) | < 0.00001 |
| Follow up time | 0.35 (-0.29, 0.99) | 0.28 |
|  |  |  |
| R-squared | 0.68 |  |
| AIC | 2444.8 |  |
| **Multiple logistic regression** | | |
| Age at treatment | 0.96 (0.90, 1.02) | 0.18 |
| Sex (female) | 1.12 (0.41, 3.07) | 0.82 |
| Education | 0.91 (0.70, 1.18) | 0.48 |
| Smoking | 1.03 (0.41, 2.59) | 0.95 |
| Radiation dose | 1.00 (0.99, 1.001) | 0.51 |
| Chemotherapy | 0.85(0.21, 3.33) | 0.81 |
| Physical fatigue (MFI scale) | 0.88 (0.75, 1.03) | 0.12 |
| Reduced activity (MFI scale) | 1.11 (0.95, 1.29) | 0.19 |
| Mental fatigue (MFI scale) | 1.32 (1.15, 1.51) | 0.82 |
| General fatigue (MFI scale) | 1.11 (0.94, 1.29) | 0.23 |
| Reduced motivation (MFI scale) | 0.88 (0.74, 1.05) | 0.15 |
| Total mood | 1.04 (1.02,1.06) | 0.26 |
| Follow up time | 1.05 (0.88, 1.26) | 0.60 |
|  |  |  |
| R-squared | 0.44 |  |
| AIC | 179.66 |  |

Stepwise selection approach was implemented in both the linear and logistic regression. Model comparisons alongside their degree of freedom, AIC, chi-square values and deviance was undertaken and final model with the lowest AIC was selected . The result from final model is presented in Table 4 below.

**Table 4**. Results from final NCC regression model

| **Multiple linear regression** | | |
| --- | --- | --- |
| **NCC** | ***Co-efficient (95%CI)*** | **P value** |
| Total mood  disturbance (POMS-SF) | -0.32 (-0.41, -0.24) | <0.001* |
| Mental fatigue(MFI scale) | -2.59 (-3.06, -2.11) | <0.001* |
| General fatigue(MFI scale) | -0.75(-1.34, -0.17) | <0.05* |
| Physical fatigue(MFI scale) | 0.65 (0.15, 1.15) | <0.01* |
| Age at treatment | 0.11 (-0.09, 0.30) | 0.28 |
| Education | 0.06 (-0.81, 0.91) | 0.90 |
| Sex (female) | -2.72 (-6.18, 0.74) | 0.12 |
|  |  |  |
| R² | 0.670 |  |
| AIC | 2428.5 |  |
| **Multiple Logistic regression** | | |
| **NCC** | ***Odds ratio (95%CI)*** | **P value** |
| Total mood disturbance (POMS-SF) | 1.04 (1.02, 1.06) | < 0.001* |
| Mental fatigue (MFI scale) | 1.31 (1.14, 1.49) | <0.001* |
| General fatigue (MFI scale) | 1.08 (0.93, 1.25) | 0.34 |
| Physical fatigue (MFI scale) | 0.93 (0.80, 1.07) | 0.28 |
| Age at treatment | 0.97(0.92, 1.03) | 0.29 |
| Education | 0.93 (0.72, 1.19) | 0.55 |
| Sex (female) | 1.04 (0.39, 2.76) | 0.93 |
|  |  |  |
| R² | 0.39 |  |
| AIC | 170.56 |  |

*statistically significant p<0.05

3.2. **ACS ( NCF)**

The Z-score for Box tapping (dependent variable) is measured as a continuous outcome for the linear regression and the deficit across all domains (dependent variable) is measures as binary outcome for the logistic regression. The independent variables included in the NCF model are age at diagnosis, smoking(yes or no), radiation dose, chemotherapy (yes or no), physical fatigue, reduced activity, mental fatigue, general fatigue, reduced motivation, total mood and follow up time. The coefficient of all variables included in the model before the stepwise selection approach was implemented is presented in table 5 below:

**Table 5.** Coefficient of all variables explored in NCF regression analysis

| **Multiple linear regression** | | |
| --- | --- | --- |
| **NCF** | ***Co-efficient (95%CI)*** | **P value** |
| Age | 0.29 (-2.20, 2.78) | 0.24 |
| Smoking | -0.022 (-0.06, 0.01) | 0.61 |
| Radiation dose | 0.00 (0.00, 0.0001) | 0.19 |
| Chemotherapy | -0.27(-1.31,0.76) | 0.61 |
| Physical fatigue (MFI scale) | 0.04 (-0.07, 0.16) | 0.46 |
| Reduced activity (MFI scale) | -0.005 (-0.13, 0.12) | 0.94 |
| Mental fatigue (MFI scale) | 0.06 (-0.04, 0.15) | 0.23 |
| General fatigue (MFI scale) | -0.005(-0.09, 0.10) | 0.92 |
| Reduced motivation (MFI scale) | -0.08 (-0.03, 0.05) | 0.23 |
| Total mood disturbance (POMS-SF) | -0.02 (-0.04, -0.002) | 0.03 |
| Follow up time | 0.04 (-0.08, 0.16) | 0.57 |
|  |  |  |
| R-squared | 0.16 |  |
| AIC | 276.96 |  |
| **Multiple logistic regression** | | |
| **NCF** | ***Odds Ratio (95% CI)*** | **P value** |
| Age | 1.02 (0.95, 1.09) | 0.58 |
| Smoking | 1.53 (0.54, 4.29) | 0.42 |
| Radiation dose | 1.00 (1.00, 1.00) | 0.63 |
| Chemotherapy | 2.03(0.25, 16.30) | 0.52 |
| Physical fatigue (MFI scale) | 0.90 (0.71, 1.12) | 0.34 |
| Reduced activity (MFI scale) | 0.97 (0.76, 1.22) | 0.78 |
| Mental fatigue (MFI scale) | 0.88 (0.73, 1.06) | 0.18 |
| General fatigue (MFI scale) | 1.08 (0.89, 1.32) | 0.42 |
| Reduced motivation (MFI scale) | 1.26 (0.98, 1.61) | 0.10 |
| Total mood disturbance (POMS-SF) | 1.02 (0.99, 1.06) | 0.15 |
| Follow up time | 0.87 (0.67, 1.12) | 0.28 |
|  |  |  |
| R-squared | 0.14 |  |
| AIC | 116.44 |  |

Stepwise selection approach was implemented in both the linear and logistic regression. Model comparisons alongside their degree of freedom, AIC, chi-square values and deviance was undertaken and final model with the lowest AIC selected. The result from final model is presented in Table 6 below

**Table 6**. Results from final NCF regression model

| **Multiple linear regression** | | |
| --- | --- | --- |
| **NCF** | ***Co-efficient (95%CI)*** | **P value** |
| Total mood  disturbance (POMS-SF) | -0.02 (-0.04, -0.006)* | <0.05* |
| Mental fatigue (MFI scale) | 0.04 (-0.04, 0.13) | 0.36 |
| General fatigue (MFI scale) | 0.001 (-0.10, 0.09) | 0.87 |
| Physical fatigue (MFI scale) | 0.003 (-0.07, 0.08) | 0.80 |
| Age at treatment | -0.02 (-0.05, 0.01) | 0.18 |
| R² | 0.15 |  |
| AIC | 271.35 |  |
|  |  |  |
| **Multiple Logistic regression** | | |
| **NCF** | ***Co-efficient (95%CI)*** | **P value** |
| Total mood  disturbance (POMS-SF) | 1.02 (1.00, 1.06) | 0.07 |
| Mental fatigue(MFI scale) | 0.91 (0.76, 1.08) | 0.26 |
| General fatigue(MFI scale) | 1.10 (0.91, 1.32) | 0.31 |
| Physical fatigue(MFI scale) | 1.07 (0.84, 1.13) | 0.70 |
| Age at treatment | 1.00 (0.94, 1.06) | 0.53 |
| R² | 0.11 |  |
| AIC | 103.84 |  |

*statistically significant p<0.05

**Multicollinearity**:

Multicollinearity was accessed in all the models. VIF was < 5, indicating no concerns for multicollinearity among the variables.

**Table 7.** multicollinearity table

| **Variable** | **NCF linear** | **NCF logistic** | **NCC linear** | **NCC logistic** |
| --- | --- | --- | --- | --- |
| Age | 1.10 | 1.12 | 1.15 | 1.13 |
| Physical fatigue | 2.05 | 2.34 | 2.42 | 2.58 |
| Mental fatigue | 2.27 | 2.29 | 1.90 | 1.44 |
| Total mood | 2.36 | 2.45 | 2.01 | 1.55 |
| General fatigue | 2.57 | 1.96 | 2.83 | 1.99 |
| Education | -- | -- | 1.02 | 1.09 |
| Sex | -- | -- | 1.03 | 1.16 |

**REFERENCES**

[1] Luijendijk M AvR, J., Lee Meeuw Kjoe, P., van der Wall, E., Vermeulen, I., & Schagen, S. Modeling Nonlinear Age Effects on Cognition: Advanced Statistical Methods for Establishing Normative Data Annual North American Meeting of the International Neuropsychological Society. New York, United States 2024.

[2] Feenstra HEM, Murre JMJ, Vermeulen IE, Kieffer JM, Schagen SB. Reliability and validity of a self-administered tool for online neuropsychological testing: The Amsterdam Cognition Scan. Journal of Clinical and Experimental Neuropsychology. 2018;40:253-73.

[3] Luijendijk MJ, Buijs SM, Jager A, Koolen SLW, van der Wall E, Schagen SB, et al. Effects of tamoxifen on cognitive function in patients with primary breast cancer. Br J Cancer. 2025;132:180-7.

[4] Stasinopoulos DM, Rigby RA. Generalized Additive Models for Location Scale and Shape (GAMLSS) in R. Journal of Statistical Software. 2007;23:1 - 46.

[5] Tohidinezhad F, Di Perri D, Zegers CML, Dijkstra J, Anten M, Dekker A, et al. Prediction Models for Radiation-Induced Neurocognitive Decline in Adult Patients With Primary or Secondary Brain Tumors: A Systematic Review. Frontiers in Psychology. 2022;13.
